# Sanitary Risks Connected to the Consumption of Infusion from *Senna rotundifolia* L. Contaminated with Lead and Cadmium in Cotonou (Benin)

**DOI:** 10.1155/2014/376503

**Published:** 2014-01-29

**Authors:** S. A. Montcho, K. Koudouvo, A. P. E. Yehouenou, P. Guedenon, L. Koumolou, M. Oke Sopoh, V. Dougnon, Mensavi F. Gbéassor, E. E. Creppy, M. Boko, A. P. Edorh

**Affiliations:** ^1^Interfaculty Centre of Training and Research in Environment for Sustainable Development (CIFRED), University of Abomey-Calavi (UAC), Jéricho, 03 BP 1463 Cotonou, Benin; ^2^Department of Physiology/Pharmacology, Faculty of Science, University of Lomé (UL), BP 1515, Lomé, Togo; ^3^Laboratory of Applied Research in Biology (LARBA), Genie Environment Department of Polytechnic University (EPAC), University of Abomey-Calavi (UAC), 01 BP 526 Cotonou, Benin; ^4^National Programme for Malaria Control (PNLP), Ministry of Health, Akpakpa, 01 BP 882 Cotonou, Benin; ^5^Laboratory of Toxicology and Applied Hygiene/UFR of Pharmaceutical Sciences, 146 Street Léo Saignat, 33076 Bordeaux Cedex, France; ^6^Laboratory Pierre Pagney: Climate, Water, Ecosystem and Development (LACEEDE), Faculty of Arts and Human Sciences, University of Abomey-Calavi, 03 BP 1122 Cotonou, Benin; ^7^Department of Biochemistry and Cellular Biology, University of Abomey-Calavi (UAC), 01 BP 526 Cotonou, Benin

## Abstract

This study carried out an assessment of sanitary risks connected to the consumption of *Senna rotundifolia* Linn. contaminated with lead and cadmium. This plant was collected and analyzed by atomic absorption spectrophotometry. The results revealed a contamination of plants from markets of Dantokpa, Vossa, and Godomey with heavy metals. *Senna* from Vossa was higher in cadmium and lead levels (Pb: 2.733 mg/kg ± 0.356 mg/kg; Cd: 0.58 mg/kg ± 0.044 mg/kg) compared to the two other places (Pb: 1.825 mg/kg ± 0.133 mg/kg, Cd: 0.062 mg/kg ± 0.015 mg/kg and Pb: 1.902 mg/kg ± 0.265 mg/kg, Cd: 0.328 mg/kg ± 0.024 mg/kg), respectively, for Dantokpa and Godomey. In terms of risk assessment through the consumption of *Senna*, the values recorded for lead were nine times higher with children and six times higher with adults than the daily permissive intake (Pb: 3.376 × 10^−2^ mg/kg/day for children and 2.105 × 10^−2^ mg/kg/day for adults versus 3.6 × 10^−3^ mg/kg/day for DPI). With respect to cadmium, there was no significant difference between the recorded values and the DPI (Cd: 1 × 14 10^−3^ mg/ kg/day for children and Cd: 0.71 × 10^−3^ mg/ kg/day for adults versus Cd: 1 × 10^−3^ mg/kg/day for adults). This exposure of the population to lead and cadmium through the consumption of antimalarial healing plants could pose public health problems.

## 1. Introduction

Air emissions of lead and cadmium are mainly anthropological [1]. Indeed, numerous authors brought to light an increase of 20 factors in connection with human activities during the last two centuries [2, 3]. And currently the main sources are still fuel combustion in urban zones and the use of fungicides in rural areas [4, 5]. This study aims at assessing the risks of exposure to lead and cadmium connected to the consumption of *Senna rotundifolia *Linn. in Cotonou. The plant* Senna rotundifolia *Linn. was chosen because it is the first plant that is the most frequently bought in markets during the ethnobotanical survey. Benin has been facing enormous problems of environmental pollution connected to the production, installations, storage, or treatment process of wastes or pollutants. This situation is amplified by the problems of purification of liquid and solid wastes, draining muds, and household and nonhousehold wastes [6] following the important urban pressure. Certainly, this situation may entail the contamination with pollutants—in this particular case toxic heavy metals of all compartments of the environment—but the ground is the main target.

Heavy metals spread up in grounds under various forms. Their forms depend on the dynamics of their mineralogical composition, salinity, pH, redox conditions, granulometry of the ground, its moisture content, and the presence of microorganisms [7]. All these factors influence the solubility of metals or their adsorption [8]. Heavy metals are persistent in the ground because they are not degradable by chemical or biological processes [7]. If they are not present in the metallic state, they can settle in clays or in organic matters by ionic connection or form complexes in solution. They can also form inorganic compounds or settle on the surface of particles by adsorption [7]. In this way, they accumulate in the ground where they are drained by run-off water in deeper layers, possibly up to groundwaters [9].

Situated in the interface between water, atmosphere, and vegetables, the ground ensures numerous functions: economic, ecological, and biological [10]. Supporting numerous human activities (industrialization, urbanization, and agriculture), its main role regarding the environment has long been admitted. The ground intervenes as a reactor, a receiver, an accumulator, and a filter of pollutions [11]. However, there was really no serious focus set on the implication of the ground in the cycle of matter and the ground was thought to absorb. On the contrary, toxic heavy metals accumulate in the ground where they may enter the food chain through vegetables [12]. The concentration in toxic heavy metals increase by time [13]. These metals may be released when the environment is altered (acidification of the ground under the influence of changes in temperature, humidity, etc.) and can thus constitute a real time threat. The first victims are the vegetables among which are healing plants.

On the other hand, there is no need to prove the presence of toxic heavy metals in the food intake of Beninese [14, 15]. Concerning Benin, the first works conducted in the field of toxicology showed that the marine environment is strongly polluted by heavy metals, as well as the water, sediments, and fishing products [16]. Agonkpahoun [17] analyzed the water, the sediments and the fishing products of Lake Nokoué and River Okpara. Studies carried out by Guedenon et al. [18] and Hounkpatin et al. [19], respectively, in Oueme river at Bonou and the lakeside city of Ganvié revealed high levels of heavy metals in continental waters of Benin. Gnandi et al. [20] reported the bioaccumulation of certain trace elements in market-gardened products cultivated on the urban grounds along the highway Lomé-Aného (south Togo).

Toxic heavy metals were found in snails [14], grounds, and vegetables [15, 21]. The risks of affecting human health were estimated for toxic heavy metals. Indeed, lead and cadmium are poisons for the metabolism.

In fact, absorption of lead depends on the physical and chemical state of the metal and is influenced by age, physiological status, nutritional status, and genetic factors [22]. In the general public, exposure to lead occurs primarily through the oral route, with some contribution from inhalation [23]. Gastrointestinal absorption of lead is affected by physicochemical characteristics of the lead particles and by physiological factors including age, fasting, nutritional calcium and iron statuses, and pregnancy [24]. In adults without occupational exposure and in older children, lead absorbed by the gastrointestinal tract comes mainly from the intake of lead from food, drink, and soil/dust. In adults, approximately 5–15% of ingested lead is absorbed in the gut whereas in children and infants absorption may be as high as 40% [25, 26]. Low levels of calcium, iron, copper, zinc, selenium, or phosphate in the diet can increase lead absorption [22, 24, 26].

As for cadmium, absorption following oral exposure is largely dependent on the solubility of its compound but also physiological and nutritional factors may modify the amount absorbed. Absorption from the gastrointestinal tract appears to be a saturable process, as the amount absorbed is decreased at higher doses. Cadmium absorption may be decreased by divalent and trivalent cations (Zn^2+^, Mg^2+^, or Cr^3+^) and increased by iron and calcium deficiencies [27]. Cadmium is widely distributed in the body bound mainly to red blood cells or high molecular weight proteins in the plasma. Cadmium is accumulated (50–70% of body burden) in the kidneys and liver, where it induces the production of metallothionein that binds approximately 80–90% of cadmium in the body [28]. There is little or no metabolism of cadmium, although it binds to various macromolecules and proteins [27]. Metallothionein is largely involved in the binding of cadmium, which is generally thought to reduce the toxicity of cadmium. In the liver, its production is sufficient to bind all cadmium accumulated. The metallothionein-bound cadmium is released from the liver into the blood where it is cleared by glomerular filtration in the kidney and taken up by the renal tubules, where the metallothionein is cleaved and cadmium is released. As a small fraction of the cadmium is absorbed from the GI tract following ingestion, most of the oral dose is excreted in the faeces.

Lead and cadmium were particularly studied because they are among the most toxic metals for man, particularly causing more or less serious neurological lesions [29].

This study aims at assessing the levels of contamination of antimalarial healing plants with heavy metals in Cotonou on the one hand and on the other hand assessing the exposure risk to lead and cadmium of the population through the consumption of these plants.

## 2. Materials and Methods

### 2.1. Study Area

**Figure 1:**
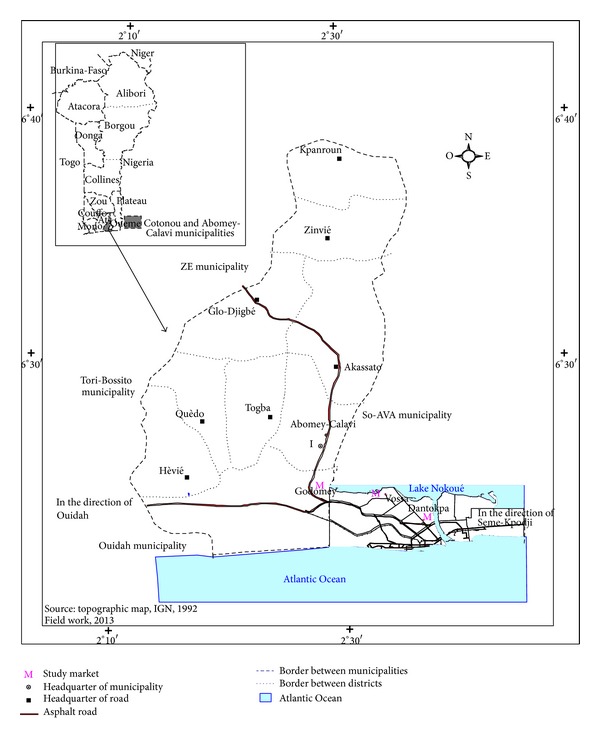
Study area location.

Cotonou is the location that served as study area (Figure 1) for our ethnobotanical study. This survey was carried out in 17 markets (the number was required by the protocol of ATRM method) and consisted in threefold purchase of antimalarial medical recipes (markets of Wologuede, Sainte-Rita, Fifadji, Zogbo, Menontin, Kindonou, Godomey, Adjatokpa, Vedoko, Casse-Auto, Vossa, Vodje-Rail, Aidjedo, Saint-Michel, Dantokpa, Gbegamey, and Fidjrosse).

### 2.2. Sampling and Heavy Metals Analyses of Antimalarial Plants

*Senna rotundifolia *Linn. was purchased in November 2010 from the three markets that concern this study and was identified by a plant taxonomist. It belongs to the Fabaceae family.

*Senna rotundifolia *Linn. was analysed for lead and cadmium by atomic absorption spectrophotometer of AAS 110.

Lead and cadmium were analyzed in the Laboratory of Sciences of Earth, Waters and Environment (LSGWE) of the National Institute of Agriculture Researches in Benin (INRAB) in Republic of Benin by flame atomic absorption spectrophotometer (SAA) 110 following the guidelines of NF reference ISO on 1146 1995 and NF X 31-147 1996. The plants were mineralized before heavy metals analyses. The data were processed with Excel.

### 2.3. Enquiry

A poll was carried out on two hundred children in the range of 10 months–5 years whose parents were used to purchasing the leaves of *Senna rotundifolia* for prevention and treatment of malaria.

### 2.4. Preparation of Decoction

The decoction was done following the guidelines by phytotherapists: 500 g of fresh leaves of *Senna rotundifolia* was boiled in 1.5 L of tap water for twenty minutes.

### 2.5. Choice of Market for Risk Assessment

Dantokpa market is the biggest market in Cotonou and receives the visit of the highest number of consumers. It even supplies other markets in medicinal plants. That is the reason it was chosen for the risk assessment survey.

### 2.6. Procedure of Risk Assessment

It is based on the method standardized by Ricoux and Gasztowtt [30].

The approach of risk assessment which is standardized by Ricoux and Gasztowtt [30] is conducted in four stages:

- selection of substances and identification of dangers;
- selection of the toxicologic reference values;
- assessment of the exposure by crossing the levels of contamination of plants with Pb and Cd and the quantities consumed by a sample of the population. The data on the habits, the quantity, and the frequency of consumption of plants were collected through opinion poll;
- last but not least, the characterization of the risk which represents the synthesis stage of the approach, the presentation, and discussion of the results related to the calculation of the danger quotient (DQ).

## 3. Results

### 3.1. Toxicological Reference Values

The safety values of daily permissive intake (DPI) by French Agency of Sanitary and Food Safety (AFSSA) [31] for cadmium and lead are, respectively, 1 × 10^−3^ mg/kg and 3.6 × 10^−3^ mg/kg per body weight (BW). Also, the permissive limits for cadmium and lead by WHO [32] in any food product meant for consumption are, respectively, 0.2 mg/kg and 0.3 mg/kg and as healing plants are considered as food, the aforementioned standard was taken into consideration.

### 3.2. Data Collection of Consumption of Plants

Table 1 summarizes the data collected with regard to levels of consumption of healing plants in the study area.

The results of the survey concerned 200 children from 10 months to 5 years old.

### 3.3. Dose

From the survey, it came up that 17% of the children whose ages ranged between 3 and 5 years old received three times the dose of 0.25 L and that dose is applicable to adults whereas children below three years old received the dose of 0.1 L.

The quantities were estimated according to the volumes of herbal teas of *Senna rotundifolia *L. adopted by parents who give these herbal teas at least once a day to their children.

The minimal mean quantity of herbal teas consumed by a child in a day is the median *M*:

(1) $$\begin{array}{c} M=\frac{\left(0.25\times 17\times 3+0.10\times 83\times 3\right)}{100}=0.37\;\text{L}. \end{array}$$

### 3.4. Measurement of Lead and Cadmium in *Senna rotundifolia* Linn

In the present study, *Senna rotundifolia *L., which is the most frequently bought species in the biggest market of Cotonou (Dantokpa), was chosen for the risk assessment.

Table 2 presents the results of lead and cadmium concentrations in *Senna rotundifolia *Linn. in three markets.

For lead, there is no significant difference between mean values with the same letter; however, there is a high significant difference between values with different letters (*P* = 0.000). The same applies to Cd.

Apart from Cd mean value recorded at Dantokpa which showed no difference with safety value, all the mean values are highly above the permissive values by WHO (*P* = 0.000).

*Senna rotundifolia *L. is contaminated with lead and cadmium and the mean concentrations were, respectively, 1.825 ± 0.133 ppm and 0.062 ± 0.015 ppm.

### 3.5. Characterization of the Risk: Calculation of the DED (Daily Exposure Dose)

For *Senna rotundifolia *Linn. contaminated with Pb and consumed by the children,

(2) $$\begin{array}{c} {\text{DED}}_{\text{Pb}}=\frac{\left(Q\times C_{\text{Pb}}\right)}{\text{BW}}, \end{array}$$

where DED_Pb_ is daily exposure dose for lead, *Q* = 0.37 L/day = 0.37 kg/day (mean quantity of *Senna rotundifolia *L. consumed by a child), *C*_Pb_ = 1.825 mg/kg = 1825 *μ*g/kg (lead mean concentration in *Senna rotundifolia *L.), and BW = 20 kg [31] (body weight of the consumer (child)).

Thus,

(3) DEDPb=(0.37 Kg/day×1825 μg/kg)20 kg=3.376×10−2 mg/kg.

For an adult of 65 kg, DED is

(4) DEDPb=(0.25 kg/day×3×1825 μg/kg)65 kg=2.105×10−2 mg/kg.

For *Senna rotundifolia *Linn. contaminated by Cd and consumed by children we have the following.

With the aforementioned formulae

 
*Q*_Cd_ = 0.37 L/day = 0.37 kg/day
 
*C*_Cd_ = 0.062 mg/kg = 62 *μ*g /kg
 BW = 20 kg [31].

Thus,

(5) $$\begin{array}{c} {\text{DED}}_{\text{Cd}}=\frac{\left(0.37\;\text{kg}/\text{day}\times 62\;\mu \text{g}/\text{kg}\right)}{20\;\text{kg}}=1.14\times 10^{-3}\;\text{mg}/\text{kg}. \end{array}$$

For an adult of 65 kg, the DED is

(6) DEDCd=(0.25 kg/day×3×62 μg/kg)65 kg=0.71×10−3 mg/kg.

In addition to DED_Cd_ and DED_Pb_ through consumption of plant tea, there could be exposure to cadmium and lead through average daily food intake (ADFI). That DED is known as DED of general feeding.

Table 3 shows the amount of lead and cadmium brought in by the average daily food intake of a child (ADFI) and the DED of the general feeding [31].

DED  child is daily dose of total exposure for a toxicant expressed. DED_PT_ is daily dose of exposure through the consumption of *Senna rotundifolia *L. contaminated with a toxicant. DED_FI_ is daily dose of exposure to a toxicant through general feeding.

Thus,

(7) for  Pb, DED  child=33.76 μg/kg/day+2.60 μg/kg/day=3.636×10−2 mg/kg/day;for  Cd, DED  child=1.14 μg/kg/day+0.38 μg/kg/day=1.52×10−3 mg/kg/day.

In theory, considering the fact that for an adult the meals are often the same within households, the DED for an adult is

(8) $$\begin{array}{c} \text{DDE}\;\text{per}\;\text{adult}=\left(\text{DED}\;\text{child}\times \text{AW}\;\text{child}\right)\text{AW}\;\text{of}\;\text{adult}, \end{array}$$

where DED  child is daily dose of exposure for child, DED  adult is daily dose of exposure for adult, and AW is average weight of an adult =65 kg.

Thus,

(9) for  Pb, adult  DED=(36.36 μg/kg/day×20)65=1.118×10−2 mg/kg/day;for  Cd, adult  DDE=(1.52 μg/kg/day×20)65=0.46×10−3 mg/kg/day.

### 3.6. Calculation of Danger Quotients

The danger quotient is defined by the relationship between the DDE observed on average and the corresponding BDD according to the formula

(10) $$\begin{array}{c} \text{DQ}=\frac{\text{total}\;\text{DED}}{\text{BDD}}, \end{array}$$

with

 DQ = danger quotient,
 total DED = daily dose of total exposure,
 BDD = bearable daily; for lead = 3.6 *μ*g/kg/day.

For children,

 DQ of lead = 36.36 *μ*g/kg/day/(3.6 *μ*g/kg/day) = 10.10,
 DQ of cadmium = 1.52 *μ*g/kg/day/(1 *μ*g/kg/day) = 1.52.

For adults,

 DQ of lead = 11.18 *μ*g/kg/day/(3.6 *μ*g/kg/day) = 3.10,
 DQ of cadmium = 0.46 *μ*g/kg/day/(1 *μ*g/kg/day) = 0.46.

Table 4 presents the main results of exposure assessment to Pb and Cd through the consumption of contaminated plants.

## 4. Discussion

The assessment of the exposure of the target population to lead and cadmium through the consumption of herbal teas made of *Senna rotundifolia *Linn. required the analysis of two sources of data in accordance with Ricoux and Gasztowtt [30] guidelines: information about the frequency of consumption of herbal tea through poll opinion (Table 1) and the results of lead and cadmium analyses in *Senna rotundifolia* with atomic absorption spectrophotometer (Table 2). The mean concentrations for lead and cadmium were, respectively, 1.825 ppm ± 0.133 and 0.062 ppm ± 0.015. These mean concentrations in the herbal tea were above WHO [32] standards which are, respectively, 0.3 ppm and 0.2 ppm. It was also noticed that *Senna rotundifolia *Linn. from Vossa was higher in cadmium and lead levels (Pb: 2.733 mg/kg ± 0.356 mg/kg; Cd: 0.58 mg/kg ± 0.044 mg/kg) compared to the two other places (Pb: 1.825 mg/kg ± 0.133 mg/kg; Cd: 0.062 mg/kg ± 0.015 mg/kg and Pb: 1.902 mg/kg ± 0.265 mg/kg; Cd: 0.328 mg/kg ± 0.024 mg/kg), respectively, for Dantokpa and Godomey. In fact, Vossa is notoriously well-known as the most unsafe place of Cotonou. It is a swampy area where pigs are errand. So inadequate freser vation of the plants could explain the highest levels of heavy metals in that place of our study area. Besides, the DED for a 20 kg child was 3.376 × 10^−2^ mg/kg for Pb and 1.14 × 10^−3^ mg/kg for Cd. These values of DED (especially for lead) were highly above the permissive values by WHO [32] which are, respectively, 3.6 × 10^−3^ mg/kg and 1 × 10^−3^ mg/kg. Also the DED obtained for a child were much higher than those with adults of 65 kg which were, respectively, 2.105 × 10^−2^ mg/kg and 0.71 × 10^−3^ mg/kg for lead and cadmium. However, the daily exposure dose recorded through the general food intake (DED_FI_) must be added to that DED recorded for children through plants infusion considering the fact that children are exposed to the same metal via other foods in the same way as the general population [24]. The average daily food intake (ADFI) and the daily doses of exposure (DED_FI_) registered through the general feeding just for lead presented in Table 3 imply that other food items are contaminated in the same way as plants. The results revealed that the daily doses of total exposure for lead and cadmium were, respectively, lower in adults (1.118 × 10^−3^ mg/kg/day and 0.46 × 10^−3^ mg/kg/day) than in children (3.636 × 10^−2^ mg/kg/day and 1.52 × 10^−3^ mg/kg/day). In every case the danger quotient is alarmingly higher in children (10.10 and 1.52) than in adults (3.10 and 0.46). So children pay a double levy to this pollution because not only are they more exposed but also their bodies are more fragile. Indeed, children are more exposed to heavy metals than adults when considering their age, weight, and their food requirement and therefore their food intake is twice higher than that of adults and by taking in food, they are taking in lead and cadmium as well [33]. Children potentially absorb more contaminants because they spend more time outside to play and are more exposed to airborne contaminants [33]. In contrast, the clearance of heavy metals from younger individuals is slower compared to adults due to their excretion system which is not so well developed [33]. As a result, the absorbed toxicants can be more harmful to children.

Moreover, there is an excess of risk for an average consumption of 0.37 L/day of contaminated herb teas from Dantokpa market. This is not the case in adults where the risk usually proves to be lower. Of course, adults are victims of contamination or poisoning with lead and cadmium but when young children are affected, their health is particularly in danger. Very small quantities of metals can impair their cognitive development and cause attention problems. In fact, when an important proportion of pollutants and particularly lead is introduced into the body, approximately 25% remain in children's bloodstream and exert their neurotoxic damage. But in comparison to adults, only 5% to 10% of the absorbed lead remains in bloodstream. The rest settles in bones and teeth [34]. The daily doses of exposure to lead via the consumption of herb teas with *Senna rotundifolia* L. that were 3.636 × 10^−2^ mg/kg/day for lead and 1.52 × 10^−3^ mg/kg/day for cadmium were higher than those via food intake which are 2.60 × 10^−3^ mg/kg/day for Pb and 0.68 × 10^−3^ mg/kg/day for Cd. It could be inferred from the comparison that the exposure to lead is more important via the consumption of the plants than the other sources of food (Table 4). Besides, the data recorded with the population of the study area can be extrapolated to all the children in the region because of the relative homogeneity of the averages of lead and cadmium contents recorded in plants [30].

The results of the actual risk assessment accord with those of Kim et al. [35] which observed that oriental herbs in Korea contained pollutants and heavy metals. This study was the first one to estimate the exposure and risk of poisoning with heavy metals according to the standard method of Ricoux and Gasztowtt [30].

## 5. Conclusion

The objective of this work was to assess the risks of exposure to lead and cadmium in connection to the consumption of *Senna rotundifolia *Linn. infusion in Cotonou. Indeed the chemical characterization of this species showed that it is contaminated with lead and cadmium in the markets with contents which exceeded the accepted standards. The risk assessment of exposure connected to the consumption of this herb tea following the standardized approach showed that there is risk of accumulation of lead and cadmium in the body with regard to the DED and DQ calculated. Considering these results and knowing as well the fatal effects of lead and cadmium on health, it is important to raise the population's awareness of the sources of potential contamination of the plant with these metals which are essentially agriculture, industry, and the mismanagement of household wastes. The possible accumulation of lead and cadmium in the blood could be a source of pathologies known as autism and cardiorespiratory depression. But to verify this hypothesis in later studies, sampling of blood from exposed population will turn out to be necessary for the revealing of toxicity biomarkers of these metals.

## Figures and Tables

**Table 1:** Results of the survey on the consumption quantity of herb teas.

Consumption frequency	Quantity (L)	Total volume/day (L)	%Children
At least 3 times/day	0.25	0.75	17
	0.10	0.30	83

**Table 2:** Content of lead and cadmium in *Senna rotundifolia *L. in three markets.

	Lead (ppm)			Cadmium (ppm)		
(WHO 1998)	0.3			0.2		
Markets	Dantokpa	Godomey	Vossa	Dantokpa	Godomey	Vossa
*Senna rotundifolia *	1.711	2.302	2.833	0.055	0.345	0.589
	1.823	2.033	3.199	0.079	0.359	0.581
	1.677	1.644	2.226	0.078	0.298	0.592
	1.986	1.763	2.6	0.051	0.315	0.639
	1.928	1.768	2.807	0.047	0.323	0.514

Mean ± SD	1.825^a^	1.902^a^	2.733^b^	0.062^a^	0.328^b^	0.583^c^
	±0.133	±0.265	±0.356	±0.015	±0.024	±0.044

**Table 3:** Daily average intake and daily doses of food exposure to lead and cadmium.

DPI and DDE/metals	Lead	Cadmium
DPI child 3–5 years	52 *μ*g/day	7.6 *μ*g/day
DED per child (20 kg)	2.60 *μ*g/kg/day	0.38 *μ*g/kg/day
DED per adult	0.68 *μ*g/kg/day	0.16 *μ*g/kg/day

**Table 4:** Summary of results for assessment of exposure to Pb and Cd.

	Q (kg/day)	Ce (mg/kg)	BW (kg)		DED (mg/kg/day)		DED total (mg/kg/day)		DQ	
			Child	Adult	Child	Adult	Child	adult	Child	Adult
Lead	0.37	1.825	20	65	3.376 10^−2^	2.105 10^−2^	3.6 10^−2^	1.118 10^−2^	10.10	3.10
Cadmium		0.062			1.14 10^−3^	0.71 10^−3^	1.52 10^−3^	0.46 10^−3^	1.52	0.46

DED: daily dose of exposure; Total DED: daily dose of total exposure; Q: mean quantity of herbal tea consumed by a child; Ce: average concentration of lead or cadmium measured *in Senna rotundifolia L*.; BW: body weight of the consumer; DQ: danger quotient.
